# Recent Progress in Semiconductor-Ionic Conductor Nanomaterial as a Membrane for Low-Temperature Solid Oxide Fuel Cells

**DOI:** 10.3390/nano11092290

**Published:** 2021-09-03

**Authors:** Yuzheng Lu, Youquan Mi, Junjiao Li, Fenghua Qi, Senlin Yan, Wenjing Dong

**Affiliations:** 1 School of Electronic Engineering, Nanjing Xiaozhuang University, Nanjing 211171, China; 2014015@njxzc.edu.cn (F.Q.); senlinyan@163.com (S.Y.); 2 Faculty of Physics and Electronic Science, Hubei University, Wuhan 430062, China; miyouquan@163.com; 3 Department of Electronic Engineering, Nanjing Vocational Institute of Mechatronic Technology, Nanjing 211306, China; misslijunjiao@163.com

**Keywords:** nanomaterials, semiconductor-ionic conductor, membrane, low temperature solid oxide fuel cells

## Abstract

Reducing the operating temperature of Solid Oxide Fuel Cells (SOFCs) to 300–600 °C is a great challenge for the development of SOFC. Among the extensive research and development (R&D) efforts that have been done on lowering the operating temperature of SOFCs, nanomaterials have played a critical role in improving ion transportation in electrolytes and facilitating electrochemical catalyzation of the electrodes. This work reviews recent progress in lowering the temperature of SOFCs by using semiconductor-ionic conductor nanomaterial, which is typically a composition of semiconductor and ionic conductor, as a membrane. The historical development, as well as the working mechanism of semiconductor-ionic membrane fuel cell (SIMFC), is discussed. Besides, the development in the application of nanostructured pure ionic conductors, semiconductors, and nanocomposites of semiconductors and ionic conductors as the membrane is highlighted. The method of using nano-structured semiconductor-ionic conductors as a membrane has been proved to successfully exhibit a significant enhancement in the ionic conductivity and power density of SOFCs at low temperatures and provides a new way to develop low-temperature SOFCs.

## 1. Introduction

**Figure 1:**
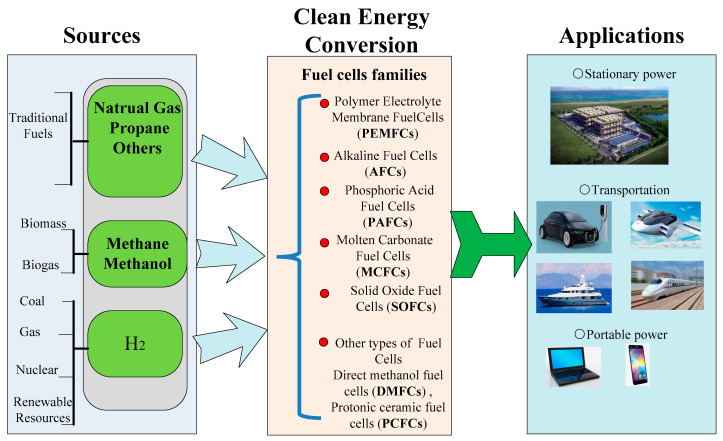
Role of fuel cells as a renewable energy resource [5].

Energy plays a vital role in social development. The consumption of traditional fossil fuels (oil, coal, and natural gas) has resulted in severe environmental pollution. With the development of society, world energy consumption is rapidly turning towards electricity. With the supply of O_2_ and fuels like H_2_, fuel cells can efficiently convert chemical energy into electricity without the release of any pollutants. As a result, it is considered one of the most ideal clean energy technologies with wide applications [1,2]. Solid Oxide Fuel Cells (SOFCs) are an important type of fuel cell among fuel cell families including Proton Exchange Membrane Fuel Cells (PEMFCs), Direct Methanol Fuel Cells (DMFCs), Phosphoric Acid Fuel Cells (PAFCs), Alkaline Fuel Cells (AFCs), Molten Carbonate Fuel Cells (MCFCs), as shown in Figure 1. Noble metal catalysts are not needed in the SOFC system due to a solid-state structure [3]. Typically, SOFCs are based on electrolytes with ionic conductivity, including oxygen-ionic conductivity, protonic conductivity, and co-ionic (H^+^/O^2−^) conductivity. A high operating temperature is required, e.g., above 800 °C, to obtain high oxygen-ionic conductivity, e.g., above 0.1 S cm^−1^ [4]. Such high temperature leads to many serious issues, i.e., high cost and insufficient lifespan, which in turn hinders the wide application of SOFCs. Therefore, decreasing the operating temperature of SOFCs becomes a hotspot issue.

Taking advantage of the superior ionic conductivity of macro-scale or nano-scale materials, the performance of SOFCs at low temperatures can be remarkably enhanced, making SOFCs an ideal candidate for clean energy conversion. Recently, Zhu’s group [6,7] found that the ionic conductivity of the membrane can be enhanced by mixing the traditional electrolytes of SOFCs with materials that exhibit electronic conductivity, like electrode materials or semiconductor materials. SOFCs using these kinds of membranes are named semiconductor-ionic membrane fuel cells (SIMFCs) [8]. It provides a new way to enhance ionic conductivity and decrease the operating temperature of SOFCs. According to reports, nanotechnology has been well used in the anode [9], cathode [10], and electrolyte layers [11] of SOFCs. However, there are still some challenges in using nanotechnology or nano-materials to develop low-temperature SOFCs [12]. This review will cover the historical development and achievement of these new SIMFCs. Then, the trends in the development of SIMFCs will be reviewed, especially the application of nanostructured materials. Finally, some recommendations about nano-SIMFCs for future work will also be discussed. 

## 2. Fundamental Concepts of Macro, Micro, Nano-Structured SOFCs and the Trend from Macro to Nano-Structured Level

### 2.1. Fundamental Concepts of Macro, Micro, and Nano-Structured SOFCs

Conventional SOFCs, with a typical structure of three layers—anode, electrolyte, and cathode—are commonly fabricated on a macro-level scale. The commercialization requires the scaling-up of cell fabrication into tens or hundreds of square centimeters in order to output power ratings in the kilowatt range. However, these cells often need to be operated at temperatures higher than 800 °C, which greatly limits the development of SOFCs and the selection of materials. To obtain improved cell performance at lower temperatures, the thickness of the cells needs to be reduced. Thin-film technologies, such as print-screening [13], sputtering [14], tape casting [15], drop coating [16] and so on, have been widely applied in the fabrication of SOFCs, enabling the reduction of electrolyte thickness. With the decrease of electrolyte thickness from hundreds of microns to tens of microns, or even several microns, the ohmic resistance of electrolytes can be remarkably reduced, resulting in the extraordinary enhancement of cell performance. For example, Shao et al. [17] achieved a peak power density of 0.7 Wcm^−2^ at 450 °C in a fuel cell constructed by micro-level electrolyte and cathode structured as Ni- Gd_0.1_Ce_0.9_O_1.95_| Gd_0.1_Ce_0.9_O_1.95_ (~14 μm)| SrCo_0.8_Nb_0.1_Ta_0.1_O_3−δ_ (~10 μm). Liu et al. [16] reported a micro-level bilayer SNDC (Sm_0.075_Nd_0.075_Ce_0.85_O_2−δ_)|ESB (Er_0.4_Bi_1.6_O_3_) film based fuel cell that showed the largest fuel cell performance (130 mW cm^−2^ @ 450 °C) among the ceria-bismuth bilayer electrolytes at operating temperatures below 550 °C. Besides, due to the inherent complexity of the internal operation of SOFCs and limitations in the experimental studies, macro-level modeling is usually taken to understand the performance as well as to improve the design. The thermodynamics, electrochemistry, and heat transfer aspects of SOFCs are usually included in the macro-level modeling [18], providing assistance for improving cell performance from experiments. However, it is relatively hard to further reduce the operating temperature while maintaining high performance. 

**Figure 2:**
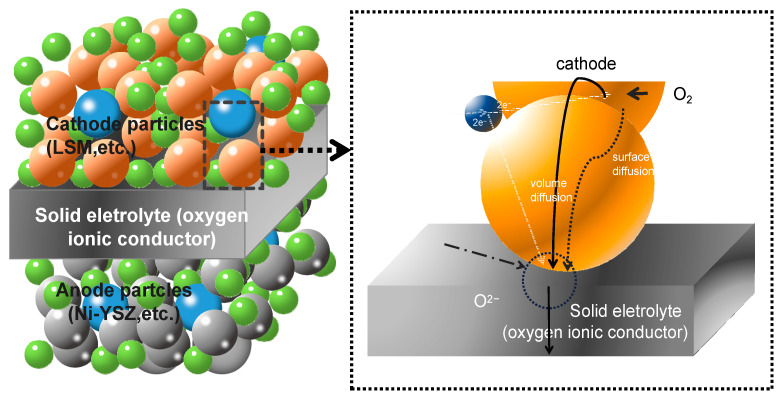
Schematic representations of molecules to ions at the triple-phase boundary [21,22].

Micro-level SOFCs were developed in 1999 [19]. In contrast to conventional SOFCs, micro-SOFCs can be operated at temperatures ranging from 700 °C to 300 °C [20], which makes it possible to be applied as a promising power source for portable electronic devices. Micro-SOFCs are usually developed based on microfabrication techniques such as thin film deposition and micropatterning. A planar micro-SOFC, which consists of electrodes and electrolytes with a layer structure, is usually fabricated on a supporting substrate that can be microstructured, i.e., Si [21]. The electrochemical stability and high performance largely depend on the mobility of ions and the microstructure of the materials as illustrated in Figure 2 [21,22]. Micro-SOFCs can achieve high performance like high specific energy (W h kg^−1^) and energy density (W h L^−1^) by using thin-film techniques [23]. For example, with a well-controlled thin-film Membrane Electrode Assembly (MEA), a typical cell with an optimized structure consisting of nano-porous Pt electrodes and 100 nm thick Y_0.16_Zr_0.84_O_1.92_ can achieve a high power density of above 1000 mW cm^−2^ at 500 °C [24]. According to their report, superior performance was attained by carefully tuning the microstructure of the porous electrode and the thickness of the electrolyte. It was also found that overall performance showed extreme sensitivity to the porosity and microstructure of the Pt anode. Using nanosphere lithography and atomic layer deposition, Printz et al. [25] fabricated a micro-SOFC with a hexagonal-pyramid array nanostructured membrane, and the cell achieved a power density of 1.34 W cm^−2^ at 500 °C. Comparing with other SOFCs, the requirement for fabrication technique is much higher in micro-SOFCs and its fabrication process is much more complex, which may result in higher cost. 

**Figure 3:**
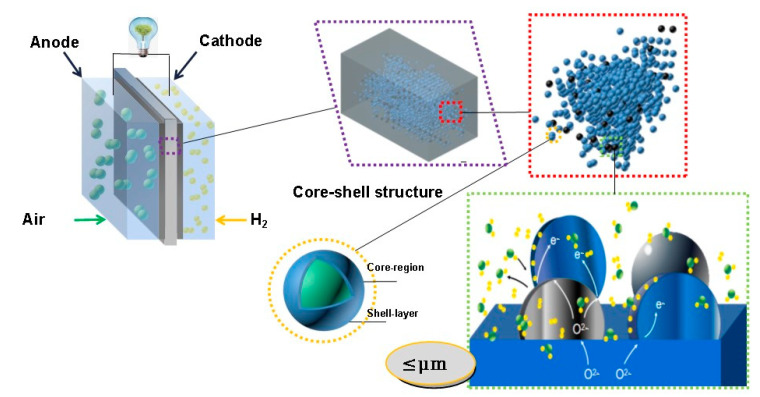
The trend of material structure from macro to nano-scale [5].

Nano-structured SOFCs are named due to the application of nanoscale materials in the components. It is well known that nanomaterials have lots of special properties that are superior to bulk materials, i.e., it has larger specific surface areas, which can provide more active sites for catalytic reactions in the electrodes [26]. Besides, when using nanomaterials in the electrolyte, surface transportation of ions can be significantly facilitated due to the formation of highly conductive surfaces and interfaces [27,28]. As a result, the development of SOFCs from macro-scale to nano-scale (Figure 3) is a promising way to improve cell performance as well as lowering the operating temperature.

According to previous reports, the properties of materials not only rely on the physical properties which can be observed at the macroscopic level but also depend on the complex interaction at the micro-scale or nano-scale [29,30], i.e., the redox reaction and nano-effect. It has been found that the structure of materials significantly impacts the performances of SOFCs [31,32]. With the rapid development of nanotechnology nowadays, techniques such as atomic layer deposition (ALD), pulsed laser deposition (PLD), sol-gel, flame spray deposition, spark plasma sintering, etc, have been applied in the fabrication of nanomaterials for SOFC applications. Microstructure engineering has become an important way to improve the performance of nanomaterials as well as cell performance. For example, the area-specific resistance (ASR) of identical composition La_0.4_Sr_0.6_Co_0.8_Fe_0.2_O_3−δ_ (LSCF) varied more than two orders of magnitude for different morphologies [33]. 

Other than electrodes, nanostructured materials including ionic conductors and semiconductors have also been successfully applied in SOFCs to reduce the operating temperature and enhance efficiency [32,34]. Recently, nanomaterials began to become a research hotspot in the SOFC field, indicating that nanomaterials and related nanotechnology will play a key role in developing low-temperature SOFCs.

### 2.2. SOFCs Based on Nano-Materials

**Figure 4:**
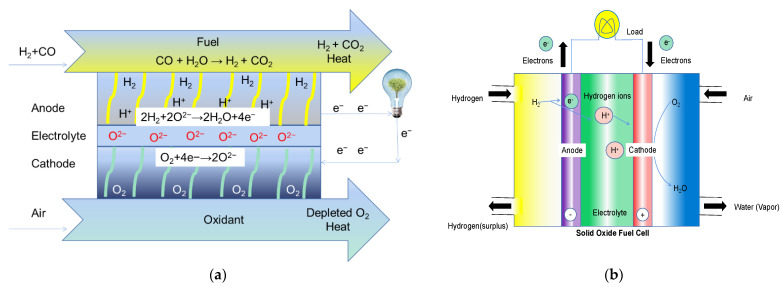
Schematic diagram of the transportation of (**a**) oxygen ions; (**b**) protons in a conventional Solid Oxide Fuel Cell (SOFC) [5].

The typical function of SOFCs with dense electrolytes and porous electrodes has been well studied. The basic reaction of a SOFC with an oxygen ionic conductor electrolyte is based on the production of oxygen ions (O^2−^) from the air reduction reaction in the cathode [35]. The as-produced O^2−^ then transport across the electrolyte layer which can also separate H_2_ from O_2_. In the anode, O^2−^ reacts with H_2_ to release electrons, which then travel to the external circuit, resulting in current output, as shown in Figure 4a. The reaction of a proton conductor-based SOFC is different. Generally, protons (H^+^) are produced from an H_2_ oxidation reaction and then transport through the dense electrolyte to react with O_2_ at the cathode to complete the reaction as shown in Figure 4b.

The operation temperature of SOFCs is mainly restricted by the electrolyte. As a result, the operation temperature can be reduced by improving electrolyte performance [26]. One effective way is to decrease the thickness of the electrolyte layer in order to reduce ion transportation resistance. Another way is to discover new materials to achieve high ionic conductivity at low temperatures. Take yttria-stabilized zirconia (YSZ) which is the most widely used electrolyte as an example, it exhibited a high ionic conductivity of 0.6 S cm^−1^ at 800 °C with a thickness of 15 nm. However, the electronic conductivity was greatly enhanced with the reduction of the thickness, resulting in a low open-circuit voltage (OCV) [36]. Therefore, searching for new methods to solve the electronic leakage problem, as well as developing new materials with high ionic conductivity at low temperatures, is raising more attention nowadays. 

**Figure 5:**
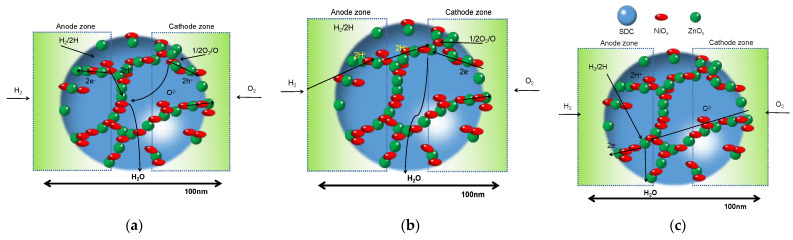
Schematic diagram of the reaction mechanism (**a**) based on O^2−^ and H^+^; (**b**) based on O and H^+^; (**c**) based on O^2−^ and H [50].

In 2011, Zhu et al. found that a single layer of the nanocomposite of LiNiZn-based oxide and doped ceria can realize the function of SOFCs which was proven to exhibit an impressive performance [37]. This new device was firstly named as electrolyte-free fuel cells (EFFCs) [37,38] or single-layer fuel cells (SLFCs) [39,40,41,42,43,44,45]. Since then, many efforts have been devoted to study this kind of fuel cell in order to understand its working mechanisms, as well as how to utilize the high ionic conductivity [46,47,48,49]. This kind of cell is a typical nano-SOFC as nanostructured materials were applied to construct the cell. Unlike the traditional three-layer SOFCs, EFFCs were believed to be based on nano-redox principles, where the reaction and ion transportation happened on the nano-scale particles. Figure 5 schematizes the reaction mechanism in fuel cells constructed based on nano-redox principles, where the transportation of protons, oxygen ions or both ions can be achieved in the nano-particles of the fuel cell. The specific redox reaction may be achieved in the following three processes [50].

(A) Completed by H^+^ and O^2−^ directly:

Hydrogen side:

H_2_ → 2H^+^ + 2e^−^, (1)

Air side:

1/2O_2_ + 2e^−^ → O^2−^, (2)

(B) Completed by H^+^ and O atom (or oxygen molecule) directly:

Hydrogen side:

H_2_ → 2H^+^ + 2e^−^, (3)

Air side:

2H^+^ + 1/2O_2_ + 2e^−^ → H_2_O, (4)

(C) Completed by O^2−^ and H atom (or hydrogen molecule) directly:

Hydrogen side:

H_2_ + O^2−^ - 2e^−^ → H_2_O, (5)

Air side:

1/2O_2_ + 2e^−^ → O^2−^, (6)

Total reaction:

H_2_ + 1/2O_2_ → H_2_O, (7)

**Figure 6:**
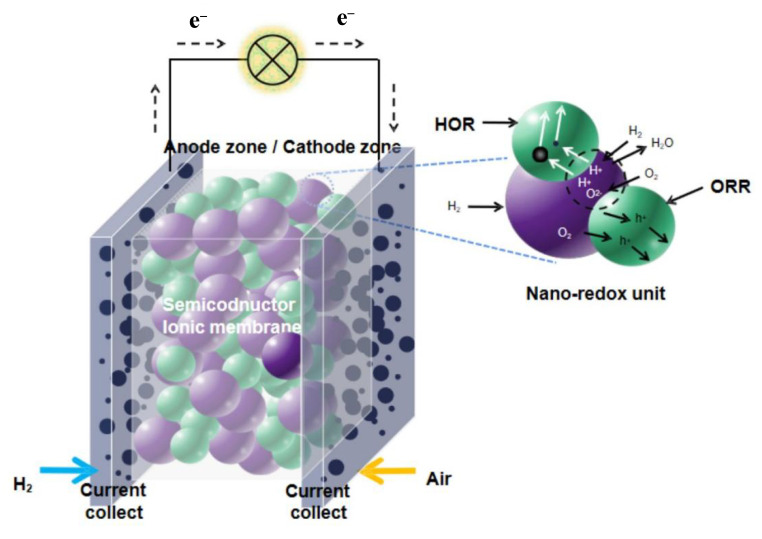
The structure of semiconductor-ionic membrane fuel cells (SIMFCs).

Recently, after having a deeper understanding of the working principle of these devices, a new name, SIMFCs [8], is given to this kind of fuel cell. SIMFC means that a mixed semiconductor-ionic conductor composite material instead of a conventional pure ionic conductor electrolyte is used as the membrane, as shown in Figure 6. Besides EFFCs (or SLFC), a kind of three-layer SIMFC is also widely studied, in which a triple conducting material, such as LiNi_0.85_Co_0.15_O_2−δ_ (LNC) [51] or Ni_0.8_Co_0.15_Al_0.05_LiO_2−δ_ (NCAL) [52], is applied on the two sides of the semiconductor-ionic membrane (SIM) as symmetrical electrodes. 

Though the structure of this three-layer SIMFC is similar to a conventional SOFC, the working mechanism of a SIMFC is a little bit different. A typical SOFC is generally based on either oxygen ion or proton transportation. However, hybrid oxygen ion—proton production and transportation are often found in a SIMFC [53,54]. On the one hand, the reducing agent (fuel) is oxidized to release electrons at the anode. On the other hand, oxygen is reduced to oxygen ion (O^2^^−^) at the cathode where oxygen combines with electrons coming from the external circuit. Then, H^+^ migrate from the anode to the cathode while O^2^^−^ transport from the cathode to the anode through the SIM layer to complete the electrochemical reaction in the device. The SIM layer mainly plays the role of ionic transportation, like the role of an electrolyte in a conventional SOFC, and may partly contribute to widening the triple-phase boundary of the cell, with the role a little different from that in an EFFC. 

## 3. Nano Materials as the Membrane of SOFCs

### 3.1. Nano-Scale Ionic Conductor as an Electrolyte

Electrolytes, which are crucial to SOFCs, are often sandwiched between the anode and cathode. Huang et al. [55] prepared a YSZ (yttria-stabilized zirconia) film with a thickness of about 50–150 nm. When it was used as the electrolyte of a SOFC, a maximum power density of 60 and 130 mW cm^−2^ was obtained at 350 and 400 °C, respectively. This result indicates that nano-scale YSZ material can be operated at temperatures as low as 350 °C with decent performance. Su et al. [56] achieved a power density as high as 677 mW cm^−2^ at 400 °C using a 70 nm YSZ membrane. However, Kosacki reported that when the electrolyte is thinner than 50 nm, its electronic conductivity will be remarkably enhanced, resulting in a sharp decrease in the open-circuit voltage [36]. Besides, it is difficult and expensive to fabricate thin-film electrolytes on a large scale, which hinders the development of thin-film SOFCs. 

Compared to the YSZ electrolyte, doped ceria exhibits higher ionic conductivity than YSZ at a lower temperature, e.g., below 600 °C. Göbel et al. [57] found that pure CeO_2_ exhibits an electronic conductivity while doped CeO_2_ holds ionic conductivity. To improve the ionic conductivity of electrolytes, elements with an atomic radius smaller than that of cerium are often selected to dope into CeO_2_. For example, rare earth elements like Sm, Gd, and Y, which are very reactive and easy to form stable compounds, are often doped or co-doped into CeO_2_. Thanks to the smaller size of the doped atoms, more oxygen vacancies can be formed when Ce atoms are replaced by these atoms, which leads to the great enhancement in the ionic conductivity. 

Among various rare earth elements doping, ceria doped with Sm (Sm_0.2_Ce_0.8_O_2−δ_, SDC) is widely investigated as it exhibits a rather high oxygen-ionic conductivity, 0.1 S cm^−1^ at 800 °C [58]. Other rare earth elements have also been reported to be successfully doped in ceria. Chen et al. [59] synthesized an epitaxial single-crystalline GDC (Gd doped CeO_2_) thin film with a thickness of 300 nm for the first time. The ionic transport activation energy (Ea) of the nano-film was 0.74 eV, demonstrating this technology is applicable for fabricating electrolytes at nanoscale with grain boundary (GB)-free property.

It is believed that proton (H^+^) conductors are superior to oxygen ion (O^2−^) conductors at lower temperatures since H^+^ conductors have lower Ea than O^2−^ conductors, and the theoretical efficiency of SOFCs based on proton conductors is higher than that of the cells based on O^2−^ [41]. In recent years, activities and efforts have witnessed the important role of proton conductors in SOFCs [60,61]. Iwahara [62], a pioneer in the study of proton conductor electrolytes, reported a proton conductor, SrCeO_3_, for application in SOFCs in the 1980s. Since then, a large number of proton conductors are reported to be applied in SOFCs, such as doped BaZrO_3_, BaCeO_3,_ and SrZrO_3_, etc. Ito et al. [63] prepared a BaCe_0.8_Y_0.2_O_3_ (BCY) thin film with a thickness of about 700 nm. A high power density of 900 mW cm^−2^ was achieved accompanied by a high open-circuit voltage of 1.1 V at 400 °C. Traversa et al. reported a recent study on a proton-conducting BaZr_0.8_Y_0.2_O_3−δ_ (BZY) ceramics, which achieved a high proton conductivity of 0.11 S cm^−1^ at 500 °C [64]. Besides, proton conduction was found in pure and doped ceria nanofilms by Gregori et al. [65]. 

In short, the application of nanomaterials and nanotechnology have greatly contributed to the development of low-temperature SOFC in lowering the activation energy of ionic conductors as well as reducing the thickness of the electrolytes, which leads to delivering optimized cell power output at low temperatures. However, there are still problems to be solved in order to further improve the performance of nano-scale ionic conductors. Firstly, the conductivity of nano-scale O^2−^ conductors is still low at low temperatures. Secondly, nano-scale H^+^ conductors show low GB conductivity and low chemical stability under fuel cell operating conditions. Additionally, the reduction of electrolyte thickness to the nanoscale has raised some other issues. For example, Guo et al. [66] found that the ionic conductivity of YSZ films with a thickness of 12~25 nm was 4 times lower than that of their microcrystalline bulk analog. Karthikeyan et al. [67] found that the chemical surface exchange rates of GDC nano-thin films were 10 times slower than those of the bulk samples in a wide range of temperatures (673–885 °C). Accordingly, it is of great importance to diminish these side-effects of nanomaterials to improve their performance. 

### 3.2. Nano-Semiconductors as a Membrane

**Figure 7:**
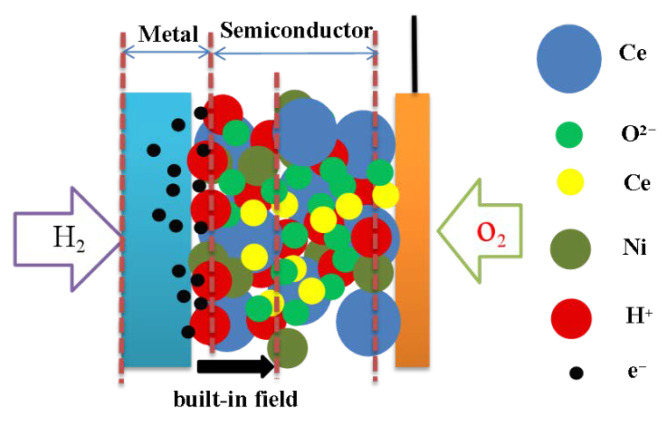
A built-in field produced by the Schottky junction [68].

Typically, semiconductor materials have been widely used in various fields like solar cells [68], photoelectronics [69], sensors [70], and so on. It has not been well applied in SOFCs due to its relatively high electronic conductivity. However, Zhu’s group found that the ionic conductivity of traditional electrolytes can be enhanced by mixing them with some electrode materials that exhibit electronic conductivity, e.g., Ni_0.8_Co_0.15_Al_0.05_LiO_2−δ_ (NCAL) [71]. It is very interesting that there is no short-circuiting issue in the cell using these composite membrane layers. On the contrary, the ionic conductivity of the semiconductor-ionic conductor composite electrolyte can be enhanced significantly compared to pure ionic conductors. Many works have been conducted to understand these issues [6,7,8,34]. Zhu et al. believed that the Schottky junction formed at the interface between the metal in the anode and the semiconductor in the composite membrane plays an important role in blocking electronic leakage [51], as shown in Figure 7. Using a single layer of the composition of a hybrid protonic-oxygen ionic conducting material Ce_0.8_Sm_0.2_O_1.9_-Na_2_CO_3_ (NSDC) and p-type semiconducting materials Co-Li co-doped NiO (LiNi_0.85_Co_0.15_O_2−δ_, LCN) with a weight ratio of 60:40, a high power density of 1000 mW cm^−2^ at 550 °C was obtained in the novel device incorporating the Schottky junction effect. This new mechanism was further verified by Zhang et al. through a composite of NCAL-NSDC (Na_2_CO_3_-SDC) membrane fuel cells [72]. A built-in field produced by the as-formed Schottky junction can enhance both proton and oxygen ion transport [51]. Dong et al. [73] believed that the high OCV (>1 V) of the SIMFC may be due to the high ionic conductivity of the SIM layer compared to its electronic conductivity. They demonstrated that SOFC can achieve a high value of OCV even when the membrane contains electronic conductivity if the ratio of ionic conductivity to electronic conductivity is high enough according to modeling, which is also the case in SIMFCs. 

**Figure 8:**
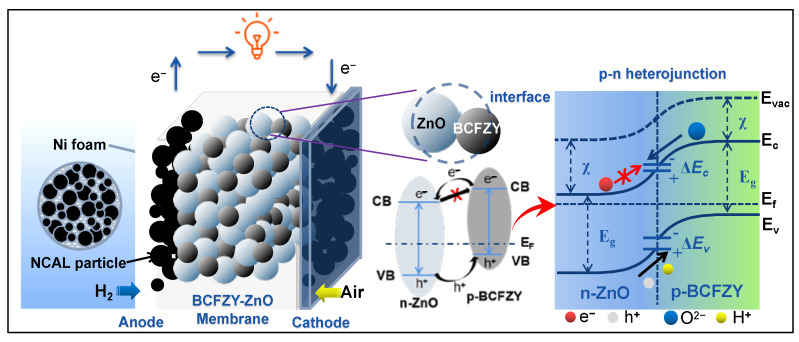
Schematic diagram of a typical p-n heterojunction formed at the heterostructure interface of the BaCo_0.4_Fe_0.4_Zr_0.1_Y_0.1_O_3−δ_ (BCFZY)-ZnO membrane layer and the corresponding energy band alignment mechanism proposed for interpreting the charge separation and ionic transportation process [74].

Inspired by the junction mechanism, Xia et al. reported a charge redistribution behavior at the particle interfaces between p-type BaCo_0.4_Fe_0.4_Zr_0.1_Y_0.1_O_3−δ_ (BCFZY) and n-type ZnO [74]. The p-n heterostructure enables charge separation by a built-in electric field (Figure 8), suppressing electron transport while promoting ionic conductivity in the meantime. 

**Figure 9:**
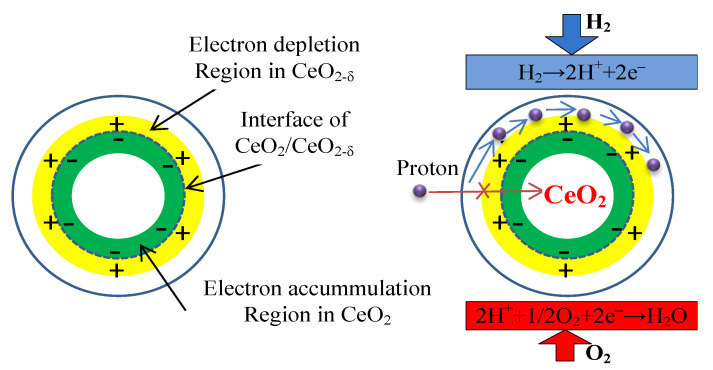
Charge separation at the interface of CeO_2−δ_/CeO_2_ particle [72].

Interestingly, Xing et al. [75] constructed a core-shell CeO_2_@CeO_2−δ_ nano-material (<100 nm) which enabled proton shuttling at the core-shell interface (Figure 9). The i-type semiconductor (CeO_2_) acted as the core and the n-type semiconductor (CeO_2−δ_) acted as the shell. At the interfaces of the CeO_2_ core and CeO_2−δ_ shell, the CeO_2−δ_ surface was positively charged while the CeO_2_ surface was negatively charged, which enabled the build-up of proton shuttles at the interfaces. This structure produced a rather high proton conductivity of 0.15 S cm^−1^. The cell that was based on this excellent proton conductor exhibited a high performance of 697 mW cm^–2^ at 520 °C. Besides, semiconductors such as SrTiO_3_ [76], TiO_2_ [77], and Li doped ZnO [78] have also been demonstrated to function as the membrane of a SOFC.

It can be seen that the conductivity of an electrolyte can be enhanced significantly by energy band design. According to physical principles, when two different semiconductors contact, band bending will happen due to the difference in the conduction band (CB) and valence band (VB) levels of the two semiconductors [79]. Xia et al. [74] supposed that electrons will move from the one with highest Fermi level to the lower one, accompanied by the bending of CB and VB until the Fermi levels of the two materials are aligned. This can result in the formation of a built-in-field (BIF), which will modulate the migration of charged carriers, i.e., electrons, holes, O^2−^, H^+^, across this space charge region. Therefore, the moving direction of electrons (or holes), as well as the migrating process of O^2−^ and H^+,^ will be affected by the formation of BIF between the two materials. In other words, if the energy band structure of SOFC is properly designed, the short-circuiting problem can be avoided, and ion transportation can be promoted as well [80,81]. 

### 3.3. Nanocomposites of Semiconductor and Ionic Conductor as a Membrane

Nanocomposite material approaches have been extensively utilized in a membrane to reduce the operating temperature of SOFCs based on already known materials or new material systems, especially composite materials of semiconductors and ionic conductors [82]. There are a number of advantages of SIMFCs compared to conventional SOFCs with three components of anode-electrolyte-cathodes. Firstly, high performance can be obtained resulting from better catalyst functions for both H_2_ and O_2_ due to the enlarged triple phase boundaries. Secondly, a faster electrochemical response can be realized as the interfaces between electrode and electrolyte are eliminated in SIMFCs. Thirdly, interface conduction is significantly improved in SIMs resulting from the hetero-interfaces of the nano-particles. With the continuous optimization of SIMFCs, high power output and ionic conductivity have been achieved. In the meantime, the long-term stability of these devices has also been improved [51]. Recently, Qiao et al. fabricated a nano-composite electrolyte by mixing semiconductor ZnO with La/Pr co-doped CeO_2_ (LCP) which is a typical ionic conductor material [53], and a maximum power density of 1055 mW cm^−2^ was achieved at 550 °C in a cell based on this composite membrane. Garcia-Barriocanal et al. [83] found that the ionic conductivity of YSZ can be enhanced significantly by forming a YSZ/STO (SrTiO_3_) nano-heterostructure. Shi et al. further provided support for improving the ionic conductivity of electrolytes through constructing semiconductor-ionic conductor heterostructures. They fabricated a SrTiO_3_/CeO_2_ heterostructure electrolyte which exhibited an ionic conductivity of 0.24 S cm^−1^ at 550 °C [84]. Wu et al. found that low-cost natural hematite material can be used as the membrane in SIMFCs. After composting it with LSCF (La_0.6_Sr_0.4_Co_0.2_Fe_0.8_O_3−δ_), a typical cathode material, a maximum power density of 467 mW cm^−2^ was achieved for the SIMFC at 550 °C. Besides, ZnO compositing with natural hematite can also improve the electrochemical performance of the SIMFC, and a power output of 580 mW cm^−2^ was achieved at 550 °C [85]. Additionally, some electrode materials, e.g., LaSrCrFe-oxides [86], LaSrCoFe-oxides [87], BaSrCoFe-oxides [88], SmFeTi-oxides [89,90], SmFe-oxides [91], CuFe-oxide [92], SrCo_0.8_Nb_0.1_Ta_0.1_O_3−δ_ [8], Sr_2_Fe_1.5_Mo_0.5_O_6-δ_ [72], LaCaMn-oxides [93], and so on, have also been proven to deliver high performance in SIMFCs. In 2020, Zhu’s group published a book named “From Electrolyte-Based to Electrolyte-free Devices” [94], providing a deep understanding of SIMFCs. Very recently, Mushaq et al. constructed a Ba_0.5_Sr_0.5_Fe_0.8_Sb_0.2_-Sm_0.2_Ce_0.8_O_2−δ_ heterostructure for SIMFCs, exhibiting a high power density of 1012 mW cm^−2^ at 550 °C [95]. Besides, other groups have also achieved good results in this field [96,97,98,99]. The most important achievements in the field of SIMFCs are listed in Table 1. In summary, there is no doubt that semiconductor materials can be used in SOFCs as a membrane. By compositing semiconductors with ionic conductors, the ionic conductivity can be remarkably improved, which is important for the SIMFC. This provides a new way for developing advanced low-temperature SOFCs.

## 4. Conclusions and Future Prospectives

Nano-scale materials have been proven to play vital roles in SOFCs. In recent decades, the development of nano-materials has paved new ways for reducing the operating temperature of SOFCs thanks to the special properties of nanomaterials that are absent in bulk phases. It overcomes the barriers faced by bulk materials while providing additional improvements in the electrocatalytic properties. Intensive research on this nanocomposite approach has also resulted in ground-breaking technologies: namely, the micro-SOFC, nano-SOFC, and SIMFC. These new fuel cells are different from the state-of-the-art fuel cells, especially nano-structured SIMFCs which replace the traditional pure ionic conductor membranes with semiconductors or the composite of semiconductors and ionic conductors. Though electronic conductors are applied in the membrane of SIMFCs, the cell can still achieve OCVs above 1 V, which is as high as that in an oxygen ion conductor-based SOFC or protonic ceramic fuel cell (PCFC) [100]. The semiconductor and ionic conductor composite can also be used to construct a single layer fuel cell in which a single layer can realize the function of both anode, cathode, and electrolyte, leading to the simplification of the structure of a SOFC. The operation temperature can be reduced significantly while holding high performance as nanostructured SIMs can deliver high ionic conductivity. Since high performance at low temperature is always the target for the development of a SOFC, it is highly necessary to develop a nanocomposite approach to lower the operating temperature of SOFCs.

Besides, more efforts should be devoted to understanding the working mechanism of SIMFCs. Firstly, it is important to investigate the fabrication of membranes by nanotechnology. Facile and cost-effective methods for manufacturing nanostructured membranes should be exploited. Secondly, new ways to construct and develop SIMFC devices, like nano-redox devices, should be studied. At the same time, in order to understand the principle of SIMFC and nano-SOFC, it is necessary to establish relevant theoretical models, like the space charge model. It is encouraged that the integration of theoretical models with experimental demonstrations can be helpful for the rational design and synthesis of novel materials with advanced or specific properties. Thirdly, particular attention should also be paid to the durability of these low-temperature SOFCs based on nanomaterials since it is one of the most critical challenges in practical applications. According to the above summarization, although high performances had been achieved in SIMFCs (above 1000 mW cm^−2^ @ 550 °C), the semiconductor-ionic membrane materials still suffer an issue from long-term operational durability. It is well believed that the high ionic conductivity of semiconductor-ionic membranes mainly comes from the interfaces or surfaces of the nanomaterials. However, due to the sintering effect, highly conductive interfaces diminish during the operation. Besides, some semiconductor-ionic membranes are not stable enough in a reduced environment, and the permeation of hydrogen into the membrane reduces the semiconductor-ionic membrane materials, leading to performance degradation. Fourthly, in order to meet the requirement of industrialization, more attention and effort should be paid to the scaling-up of the devices. Most of the samples fabricated in the lab are in a small size which does not meet the demands of industrialization, whereas when the samples are enlarged, the performances of the cells might decrease. In addition, advanced characterization techniques, e.g., in situ synchrotrons XRD (X-ray diffraction), NMR (nuclear magnetic resonance), 3D X-ray tomography microscopy, focused ion beam scanning electron microscopy, electrostatic force microscopy and spectroscopy, and isotope effect studies, etc., should be used to analyze nano- and hetero-structures, ion migration, intrinsic oxygen vacancies/defects, GB/interface/surface chemistry, coupling action between electrons and ions. These help to understand the charge-transport mechanism and detailed reaction mechanism of the nano-structured SOFCs.

Finally, new SOFC technology still faces challenges in terms of scaling-up, and long-term stability. We hope this short review can give an in-deep understanding of the nano-SOFC and SIMFCs and inspire new ideas for developing low-temperature SOFCs.

## Figures and Tables

**Table 1:** Summary of initial and recent achievements in the field of SIMFCs.

Scientist (s)	Year	Membrane Materials	Achievements	Ref.
Zhu B, Raza R, et al.	2011	Li_0.15_Ni_0.45_Zn_0.4_ -oxide (LNZ)-Sm^3+^ doped ceria	600 mW cm^−2^ @550℃	[37]
Zhu B, Raza R, et al.	2011	LiNiCuZnFeOx–Ce_0.8_Sm_0.2_O_1.9_-Na_2_CO_3_	700 mW cm^−2^ @550℃	[39]
Xia YJ, et al.	2012	Ce_0.8_Sm_0.2_O_2−δ_-Li_0.15_Ni_0.45_Zn_0.4_	10 × 10^−2^ S cm^−1^ at 600 ℃	[96]
Zhu B	2012	Research highlight	-	[40]
Zhu B, Lund P, et al.	2013	nano-NiZn oxide-Sm_0.2_Ce_0.8_O_2__−δ_	-	[80]
Zhu B, Fan L, et al.	2014	Gd doped ceria-KAlZn-oxide (GDC–KAZ) and the LiNiCuZn-oxide (LNCZ)	628 mW cm^−2^ @580 ℃	[6]
Dong X, et al.	2014	Ce_0.8_Sm_0.2_O_2−δ_–Na_2_CO_3_-Sr_2_Fe_1.5_Mo_0.5_O_6−δ_	360 mW cm^−2^ @550 ℃	[97]
Zagórski K, et al.	2014	BaCe_0.6_Zr_0.2_Y_0.2_O_3–δ_ and L_i2_O:NiO:ZnO	8 × 10^−4^ S cm^−1^ at 572 ℃	[98]
Zhu B, Lund P, et al.	2015	LiNi_0.85_Co_0.15_O_2−δ_-Ce_0.8_Sm_0.2_O_1.9_-Na_2_CO_3_	1080 mW cm^−2^ @550 ℃	[51]
Zagórski K, et al.	2017	BaCe_0.6_Zr_0.2_Y_0.2_O_3−δ_ and (Li_2_O, NiO, ZnO)	3.86 mW cm^−2^ @600 ℃	[99]
Zhu B, Huang Y, et al.	2016	La_0.2_Sr_0.25_Ca_0.45_TiO_3−δ_-Sm_0.2_CaCe_0.8_O_2__−δ_	1080 mW cm^−2^ @550 ℃	[81]
Zhu B, Wang B, et al.	2017	La_0.6_Sr_0.4_Co_0.2_Fe_0.8_O_3−δ_- Sm and Ca co-doped ceria	1000 mW cm^−2^ @550 ℃	[87]
Fan L, Zhu B, et al.	2018	Reviewer	-	[26]
Xia C, Mi YQ, et al.	2019	BaCo_0.4_Fe_0.4_Zr_0.1_Y_0.1_O_3−δ_ -ZnO	775 mW cm^−2^ @550 ℃	[74]
Zhu B, Raza R, et al.	2020	book	-	[94]
Mushaq N, Lu Y, et al.	2021	Ba_0.5_Sr_0.5_Fe_0.8_Sb_0.2_-Sm_0.2_Ce_0.8_O_2__−δ_	1012 mW cm^−2^ @550 ℃	[95]
